# Antigen discovery by bioinformatics analysis and peptide microarray for the diagnosis of cystic echinococcosis

**DOI:** 10.1371/journal.pntd.0011210

**Published:** 2023-04-12

**Authors:** Gherard Batisti Biffignandi, Ambra Vola, Davide Sassera, Saeid Najafi-Fard, Maria Angeles Gomez Morales, Enrico Brunetti, Antonella Teggi, Delia Goletti, Linda Petrone, Francesca Tamarozzi

**Affiliations:** 1 Department of Clinical-Surgical, Diagnostic and Pediatric Sciences, University of Pavia, Pavia, Italy; 2 Department of Biology and Biotechnology “L.Spallanzani”, University of Pavia, Pavia, Italy; 3 Microbiology and Virology Unit, Diagnostic Medicine Department, IRCCS San Matteo Hospital Foundation, Pavia, Italy; 4 Translational Research Unit, National Institute for Infectious Diseases (INMI) “Lazzaro Spallanzani”-IRCCS, Rome, Italy; 5 Foodborne and Neglected Parasitoses Unit, Department of Infectious Diseases, Istituto Superiore di Sanità, Rome, Italy; 6 Department of Infectious and Tropical Diseases, Sant’Andrea Hospital University of Rome "Sapienza", Rome, Italy; 7 Department of Infectious-Tropical Diseases and Microbiology, IRCCS Sacro Cuore Don Calabria Hospital, Negrar di Valpolicella, Verona, Italy; The First Affiliated Hospital of Xinjiang Medical University, CHINA

## Abstract

**Background:**

Cystic echinococcosis (CE), caused by *Echinococcus granulosus* sensu lato, is a neglected zoonosis. Its diagnosis relies on imaging, supported by serology, while only imaging is useful for staging and follow-up. Since diagnostic tools and expertise are not widely available, new accurate and easily implementable assays for the diagnosis and follow-up of CE are highly needed.

**Methodology/Principal Findings:**

We aimed to identify new *E*. *granulosus* antigens through a bioinformatics selection applied to the parasite genome, followed by peptide microarray screening and validation in ELISA, using independent panels of sera from patients with hepatic CE and clinically relevant controls.

From 950 proteins selected *in silico*, 2,379 peptides were evaluated by microarray for IgG reactivity and eight candidates selected for validation. Reactivity to one peptide was significantly higher in the CE group (p = 0.044), but had suboptimal diagnostic accuracy.

**Conclusions/Significance:**

Here we performed bioinformatics analysis and peptide microarray for antigen discovery, useful for the diagnosis of CE. Eight candidates were selected and validated. Reactivity to one peptide associated to CE but had suboptimal diagnostic accuracy. Importantly, the database developed in this study may be used to identify other antigenic candidates for CE diagnosis and follow-up.

## Author summary

Cystic echinococcosis (CE) is a complex, neglected parasitic zoonosis. Tools and expertise in the diagnosis of CE are not widely available and the development of accurate and easily implementable diagnostic assays for CE has been highlighted as a priority also by the WHO. Antigen discovery for the immunodiagnosis of CE has relied so far on classical methods, which are impractical and poorly sensitive. Here, starting from the *Echinococcus granulosus* sensu stricto genome, we aimed to identify antigenic peptides having a potential for CE diagnosis based on a multiple-criteria strategy involving the bioinformatics selection of proteins, followed by screening by peptide microarray and validation by ELISA, using a panel of well-characterized sera from patients with hepatic CE in all development stages, and from clinically relevant controls. This methodology has virtually never been applied in the field of CE. Importantly, the developed database can be used to identify other *E*. *granulosus s*.*s*. antigenic candidates.

## 1. Introduction

Cystic echinococcosis (CE) is a parasitic zoonosis caused by infection with the larval stage of the tapeworm *Echinococcus granulosus* sensu lato (s.l.). The parasite’s life cycle develops between canids, mainly the domestic dog, as definitive hosts, and ungulates, mainly livestock such as sheep, as intermediate hosts. Humans can acquire infection by accidental ingestion of parasite eggs shed through infected dogs’ feces, and act as dead-end intermediate hosts. In humans, the parasite develops in the form of fluid-filled, expansive cysts, mainly localized in the liver, followed by lungs, but any organ and tissue can be affected [1]. CE is a chronic infection, the clinical manifestations of which are non-specific and range from asymptomatic infection to disabling and even fatal disease [1]. CE is especially prevalent in livestock-breeding communities worldwide, with the highest prevalence occurring in Central Asia, Western China, South America, East Africa, Eastern Europe and the Mediterranean. It is (under)estimated that over 1.2 million people are affected worldwide at any given time, and the World Health Organization (WHO) has recently renewed the focus on CE in its 2021–2030 roadmap for neglected tropical diseases [2]. Among the needed actions indicated for CE, the WHO highlights the improvement of diagnostics as critical. Robust and easy to apply laboratory assays for screening and diagnosis of human infection are lacking. Techniques currently employed for the diagnosis and staging of CE are not always easily implementable and require experience for their interpretation, resulting in misdiagnosis and/or inappropriate management [3], with attendant risks and costs.

The diagnosis of CE currently relies on imaging, especially ultrasound for the abdominal localizations, but instruments and specific expertise for a correct diagnosis are not widespread. Furthermore, CE cysts pass though different stages, named CE1 to CE5 according to the WHO Informal Working Group on Echinococcosis [IWGE] classification [4]. CE1, CE2, CE3b stages are active (biologically viable), CE3a transitional (a proportion biologically viable and a proportion not viable), and CE4-CE5 stages are inactive (biologically with low viability or not viable). Each stage has peculiar imaging features [4,5]. Briefly, on ultrasonography, CE1 cysts are fluid-filled unilocular cysts with double cyst wall; CE2 are characterized by the presence of fluid-filled daughter cysts inside the original cyst; CE3a present the parasitic layers of the cyst wall detached and floating in the fluid-filled cyst content; CE3b contain both daughter cysts as well as a solid component showing the features of the CE4 stage; CE4 cysts have a solid content inside which folded parasitic layers, are visible as hypoechoic structures; and CE5 cysts have the features of CE4 cysts and evident peripheral calcifications.

This heterogeneity of presentations makes the differential diagnosis of CE cysts particularly broad, and thus difficult to make outside specialized centers. At present, serology has a complementary role, supporting diagnosis when imaging is inconclusive, but seroassays are not standardized, have suboptimal specificity, and lack sensitivity especially for cysts in CE1 and CE4-CE5 stages and in extra-hepatic localizations [6–8]. Furthermore, serology is currently not useful for the follow-up of CE since it does not correlate with stage/viability of the cysts [9], which is pivotal information to choose the clinical management approach and evaluate cyst’s evolution over time. Consequently, patients have to undergo years-long follow-up by imaging to evaluate the progression of the cyst and long-term response to treatment, with attendant costs and often need of traveling to referral centers, which is often unfeasible.

The development of new laboratory-based, easy to use, and robust tests with improved performances for the diagnosis and follow-up of CE are therefore highly needed. Previous works suggested that patients with CE might have both infection-specific and stage-specific serology profiles [10–12], indicating that the development of such tests is possible. So far, 2D gel electrophoresis of cyst fluid followed by immunoblot with sera from patients with CE has been used to identify immunogenic proteins [10]. However, this method requires the collection of echinococcal cyst material from slaughtered animals or invasively from humans, which is impractical and prevent the use of material from all cyst stages, since not all cyst stages require treatment with invasive procedures. Furthermore, this approach has low sensitivity and thus might not be able to identify weakly expressed and/or stage-specific antigens. Available data on the proteomic and transcriptomic profiles of *E*. *granulosus* s.l. [13], together with the publication of its genome [12], now allow different antigen discovery approaches, such as studies based on bioinformatics, which could overcome the need for the availability of parasitic material. Antigen identification through protein/peptide microarrays challenged with patients’ sera is an innovative technology, which has greatly expanded in the last decade, also in the field of parasitic diseases, but is almost unexplored for CE [14–16]. In this study, we aimed to identify antigens of *E*. *granulosus* sensu stricto (s.l.) with potential high diagnostic accuracy and stage-specific expression, through a first *in silico* bioinformatics selection, followed by reactivity screening by peptide microarray and ELISA validation using sera from well-characterized patients with hepatic CE in different stages and clinically relevant controls with non-parasitic focal liver lesions.

## 2. Methods

### 2.1. Ethics statement

The Ethics Committee of Istituto di Ricovero e Cura a Carattere Scientifico (IRCCS) San Matteo Hospital Foundation (Pavia, Italy) (approval number: P-20180022622), National Institute for Infectious Diseases (INMI) “Lazzaro Spallanzani”-IRCCS (Rome, Italy) (approval numbers: 16/2018, 146/2020) and Sant’Andrea Hospital (Rome, Italy) (approval number: 436/11) approved the study. Samples were frozen stored sera from patients attended at IRCCS San Matteo Hospital Foundation (Pavia, Italy), at INMI “Lazzaro Spallanzani”-IRCCS (Rome, Italy), and at Sant’Andrea Hospital (Rome, Italy), who had signed the consent for research purposes.

### 2.2. Study populations

Two cohorts of CE patients and controls were included in the study: a “screening cohort” and a “validation cohort”. Demographic and clinical information of all potentially eligible patients and their samples were retrieved from clinical records. For CE patients, the etiological diagnosis of CE was based on the identification by ultrasound of pathognomonic features in liver cysts. CE cysts were staged according the WHO-IWGE classification [4]. Patients were classified in five clinical groups based on cyst stage: CE1 (active, unilocular fluid-filled cysts), CE2/CE3b (active, with daughter cysts), CE3a (transitional, with detached parasitic layers), CE4/CE5 (inactive, solid content) having attained the inactive stage as the result of therapy in the last 5 years (“CE4/CE5 therapy in the last 5 years”), and CE4/CE5 having reached inactivation spontaneously (“CE4/CE5 no therapy”). Only patients with CE of the liver as the only cyst localization were included. Patients with >1 hepatic CE were included only if the cysts in active stages were in the same stage; when both active and inactive cysts were present, the patient/sample was classified according to the stage of the active cyst(s). For the purpose of patient/sample classification, CE3a cysts were considered as active. The exclusion criteria were the presence of extra-hepatic cysts or the presence of >1 active hepatic cysts in different active stages. Controls were patients with non-parasitic focal liver lesions that could enter in differential diagnosis with CE (e.g. biliary cysts, neoplasms). The “screening cohort” and the “validation cohort” had the same inclusion/exclusion criteria.

### 2.3. Proteins localization and epitope prediction

Three software were used in parallel: TMHMM2 [17] was applied for the prediction of transmembrane helices, SignalP4 [18] to identify putative secreted proteins, and predGPI [19] to assess glycosylphosphatidylinositol (GPI)-anchored proteins. The immune system accessibility has been associated with the proteins exposed on the cell surfaces or secreted/excreted [20], all proteins that have been shown to be involved in host-immunity interactions [21]. Proteins that contain a signal peptide, as well as transmembrane proteins are accessible to the immune system and involved in several processes such as cell signaling and recognition [20]. Moreover, the GPI-anchored proteins face the extracellular environment and have different functions including surface antigens, host-pathogen immune modulation and signaling, due to their localization on the external surface of the cells [19]. Specifically for cestode parasites, membrane proteins have been shown to be promising diagnostic antigens for cysticercosis [22].

While it is still unknown which and how *E*. *granulousus*-derived molecules leave the cyst [23], based on the above background, a bioinformatics pipeline was used to detect and rank putative exposed and immunogenic peptides in the *E*. *granulosus* G1 genotype (*E*. *granulosus* sensu stricto) genome [12], considering as key parameters the presence of transmembrane (TM) domains, a signal peptide (SP), and GPI anchors.

The identification of these proteins was accomplished through the first part of the pipeline, using in parallel the TMHMM2, SignalP4 and PredGPI software, largely used in previous studies with overlapping aims to our research [14,24,25]. The results from each software were filtered as follows. The candidates detected by TMHMM2 were selected according to strong indicators of the presence of a transmembrane protein or signal peptide: the number of predicted transmembrane helices (predTMHs) was ≥ 3; the expected number of amino acids present within the transmembrane helices (ExpAAsTMHs) was > 18; the expected number of amino acids within transmembrane helices in the first 60 amino acid (First60AAs) was ≥ 10. SignalP4 results were filtered selecting proteins with D-score >0.8. The D-cutoff score is a parameter that combines both signal-peptide and cleavage site prediction networks. A score above the specified threshold (0.45) indicates the presence of a signal peptide. Finally, proteins screened with PredGPI were selected only if the score of the prediction was >99%. Gene function and subcellular localization of the candidate proteins were also checked, using NCBI database and the DeepLOC algorithm [26] respectively, to provide additional clues on protein localization.

The selected proteins were then subjected to the second step of the pipeline, with the aim to identify 20 amino acid (aa)-long peptides [27–29] with predicted B-cell antigenic properties. To accomplish this goal, three software were applied in parallel: Bepipred, SVMTrip, and LBtope. Bepipred [30] combines the predictions of a hidden Markov model and the hydrophilicity property of amino acids. This software has quite low sensitivity and high specificity, achieving a level of 80% of specificity when the sensitivity was 30%. SVMTrip [29], based on a Support Vector Machine (SVM) which combines the tri-peptide similarity assessed with the Blosum62 matrix and the Propensity scores of the tri-peptide model, showed 80% sensitivity and 55% prediction with a five-fold cross-validation. LBtope [31] is also based on a SVM algorithm including characteristics like dipeptide composition and amino acid pair (AAP) profile. These profiles are used by the software to convert the protein sequence into numerical values and then used to feed the SVM model to assess the epitope prediction [28]. Since the software Bepipred works on different frames of length, 20aa antigenic sequences were extracted from the output of this software using a Python script. All sequences defined as immunogenic were retained, using default cut-offs for Bepipred and SVMTrip, and scores above 95% motif and LBtope_confirm (confirm dataset, trained on epitopes verified at least by two studies) for LBTope.

The resulting list of antigenic candidates was then filtered using CD-HIT v4.7 [32] using sequence identity threshold of 0.7, to avoid redundancy among peptide sequences. Finally, the Parker algorithm [33] was applied to calculate the solubility index for each peptide.

### 2.4. Microarray

A custom array containing the 2,400 peptide sequences was produced by PEPperPRINT (PEPperPRINT GmbH, Heidelberg, Germany). Briefly, peptide sequences were printed in duplicate (4,800 peptide spots) onto a custom peptide microarray with additional influenza virus hemagglutinin HA (YPYDVPDYAG, 58 spots) and poliovirus (KEVPALTAVETGAT, 58 spots) control peptides. Serum samples from the “screening cohort” were diluted 1:100 and incubated for 16 hours at 4°C under gentle agitation. Reactivity was visualized using a LI-COR Odyssey Imaging System with scanning offset 0.65 mm and a 21 μm resolution. Quantification of spot intensities and peptide annotation were based on the 16-bit grayscale.tiff files at scanning intensities of 7/7. Microarray image analysis was done with an appropriate analyzer. A maximum spot-to-spot deviation of 40% was tolerated; otherwise, the corresponding intensity value was zeroed.

### 2.5. Peptides

Peptides were synthetized by CRIBI-Centro di Biotecnologie Università di Padova and purified at ≥95% purity and re-suspended in Dimethyl sulfoxide (DMSO).

### 2.6. ELISA

The validation of the microarray results was done by a home-made ELISA. The initial set-up of the methodology was performed using a pool of sera obtained from patients with CE independently of the cysts status or previous therapy (positive pool) and a pool of sera obtained from subjects with non-parasitic hepatic cysts and healthy donors (negative pool). Different concentrations of antigens, blocking buffers, dilutions of pooled sera and of secondary antibody as well as different incubation times were tested. Briefly, the optimized protocol used in the validation analysis was as follows: 5 μg of peptide diluted in phosphate buffer (PBS, pH 7.4) (Euroclone, ECB4004L) were absorbed onto Maxisorp microtiter 96-well plates (NUNC) at 4°C for 16–20 hours. Then, the plate was saturated to avoid non-specific binding for 1 hour at 37°C hour using 5% non-fat dry milk (Biosigma, 711160) in PBS-Tween20 0.05% (blocking buffer). After the blocking step, the plate was incubated for 3 hours with 100 μl of serum diluted 1:2 in blocking buffer. The specific peptide-antibody complexes were identified using a rabbit anti-human IgG conjugated to horseradish peroxidase (Merk Life Science, AP101P) diluted at 1:10,000 in blocking buffer for 1 hour at room temperature. Tetramethylbenzidine ultrasensitive (Merk, T4444) was used as a substrate for 30 minutes at room temperature. The reaction was stopped by adding 50 μl/well of 1M sulphuric acid and optical density (OD) was read by a plate reader (Biorad) at 450 nm. Washing steps were done with 300 μl/well of PBS containing 0.05% Tween20. Native Antigen B (AgB) [34–38] and an AgB peptide pool [36] were used as experimental positive controls. The OD read for the wells where no antigen was adsorbed (“peptide-/serum+/secondary antibody+”) was considered as the background. Technical negative controls were “peptide+/serum-/secondary antibody+” and “peptide-/serum-/secondary antibody+”. Results of each well were expressed as OD read value subtracted of the respective background.

### 2.7. Statistical analysis

The analysis of microarray reactivity was performed selecting the most intense spots, considering sum of spot intensities as follows: i) CE cysts subset >100 ii) active CE cysts subset >50. To determine which peptides could better work as CE predictors, all the selected spot intensity values from the microarray were analyzed through logistic regression models using R software v3.6.3 [39]. Two sets of statistical analyses were performed, first including all CE cases (n = 50) vs controls (n = 25) and then including only active (n = 30) vs inactive (n = 20) CE cysts.

Selected peptides were screened using BLAST v2.2.31 against the non-redundant (nr) database and results were manually analyzed to evaluate the potential cross-reactivity with peptides from other parasites.

The analysis of peptide reactivities measured by ELISA was carried out with Prism 8 software (Graphpad Software 8.0, San Diego, MO, USA). Medians and interquartile ranges (IQR) were calculated for continuous measures; pairwise comparisons were performed using the Mann–Whitney U test with Bonferroni correction, comparisons among groups were performed using the Kruskall-Wallis test. Sensitivity and specificity were assessed using the Receiver Operating Characteristic (ROC) curve using the “pROC” package of R [40] or Prisma software. ROC curve for evaluating the diagnostic performance of peptides during validation was calculated by using Prisma software and properly selecting controls and cases values, applying the Wilson/Brown method with a 95% confidence interval. Statistical significance was set at p<0.05 or at p deriving from Bonferroni correction.

## 3. Results

### 3.1. Study population

The screening cohort comprised sera from 75 subjects, 50 CE patients (n = 10 per each stage group) and 25 controls with non-parasitic focal liver lesions. The validation cohort included sera from 29 patients with CE classified as follows: CE1, n = 2; CE2/CE3b, n = 8; CE3a, n = 3; CE4/CE5 (therapy in the last 5 years), n = 6; CE4/CE5 (no therapy), n = 10; and 14 controls with non-parasitic focal liver lesions. Demographic and clinical information of the subjects are shown in Table 1.

**Table 1:** Demographical and clinical characteristics of the screening and validation cohorts.

	**Screening cohort**							
	**CE**					**Controls**		**P value**
	**CE1**	**CE2/CE3b**	**CE3a**	**CE4/CE5 (therapy in the last 5 years)**	**CE4/CE5 (no therapy)**			
**N (%)**	**10 (100)**	**10 (100)**	**10 (100)**	**10 (100)**	**10 (100)**	**25 (100.0)**		**-**
**Median age years (IQR)**	54 (30–63)^#^	44 (35–57)	42 (27–54) ^##^	42 (31–55)	56 (45–61)	61 (45–69)		0.08^†^
**Female sex N (%)**	8 (80.0)	2 (20.0)	8 (80.0)	5 (50.0)	8 (80.0)	18 (72.0)		**0.02** ^‡^
**Serology positive results N (%)**	3 (33.3)*	8 (80.0)	7 (77.8)*	8 (80.0)	2 (20.0)	0 (0)		**<0.0001** ^‡^
**Previous treatment N (%)**	1 (14.3)^¶^	3 (30)	2 (33.3)^§^	10 (100)	0 (0)	-		**<0.0001** ^‡^
**Current treatment N (%)**	2 (28.6) ^¶^	1 (10)	0 (0) ^§^	1 (10)	0 (0)	-		0.32^‡^
**Cysts number median (IQR)**	2 (1–2)	1 (1–2)	1 (1–1)	1 (1–1)	1 (1–1)	1 (1–2)^¶ ¶^		0.08^†^
	**Validation cohort**							
	**CE**					**Controls**		**P value**
	**CE1**	**CE2/CE3b**	**CE3a**	**CE4/CE5 (therapy in the last 5 years)**	**CE4/CE5 (no therapy)**			
**N (%)**	**2 (100)**	**8 (100)**	**3 (100)**	**6 (100)**	**10 (100)**		**14 (100)**	
**Median age years (IQR)**	69 (66–71)	51 (36–68)	48 (36–51)	44 (31–61)	49 (39–58)		62 (57–70)	0.03^†^
**Female sex N (%)**	2 (100.0)	1 (12.5)	2 (66.7)	3 (50.0)	8 (80.0)		13 (92.9)	**0.0034** ^‡^
**Serology positive results N (%)**	1 (50.0)	7 (87.5)	3 (100.0)	4 (66.7)	3 (30.0)		0(0)	**<0.0001** ^‡^
**Previous treatment N (%)**	0 (0)	3 (37.5)	2 (66.7)	6 (100.0)	2 (20.0)		-	**0.016** ^‡^
**Current treatment N (%)**	0 (0)	0 (0)	0 (0)	0 (0)	0 (0)		-	-
**Cysts number median (IQR)**	3 (3–3)	1 (1–1)	1 (1–4)	1 (1–2)	1 (1–2)		1 (1–2)^%^	0.16^†^

CE: Cystic echinococcosis; N: Number; IQR: Interquartile Range; y: year

^#^ data available for 7/10 patients

^##^ data available for 6/10 patients

* performed in 9/10 patients

^¶^ data available for 7/10 patients

^§^ data available for 6/10 patients

^¶¶^ 3 control subjects had multiple cysts and were excluded from median calculation

^%^ data available for 5/14 subjects.

^†^ Kruskall-Wallis test

^‡^ Chi square test.

### 3.2. Bioinformatics pipeline and peptide selection

**Fig 1:**
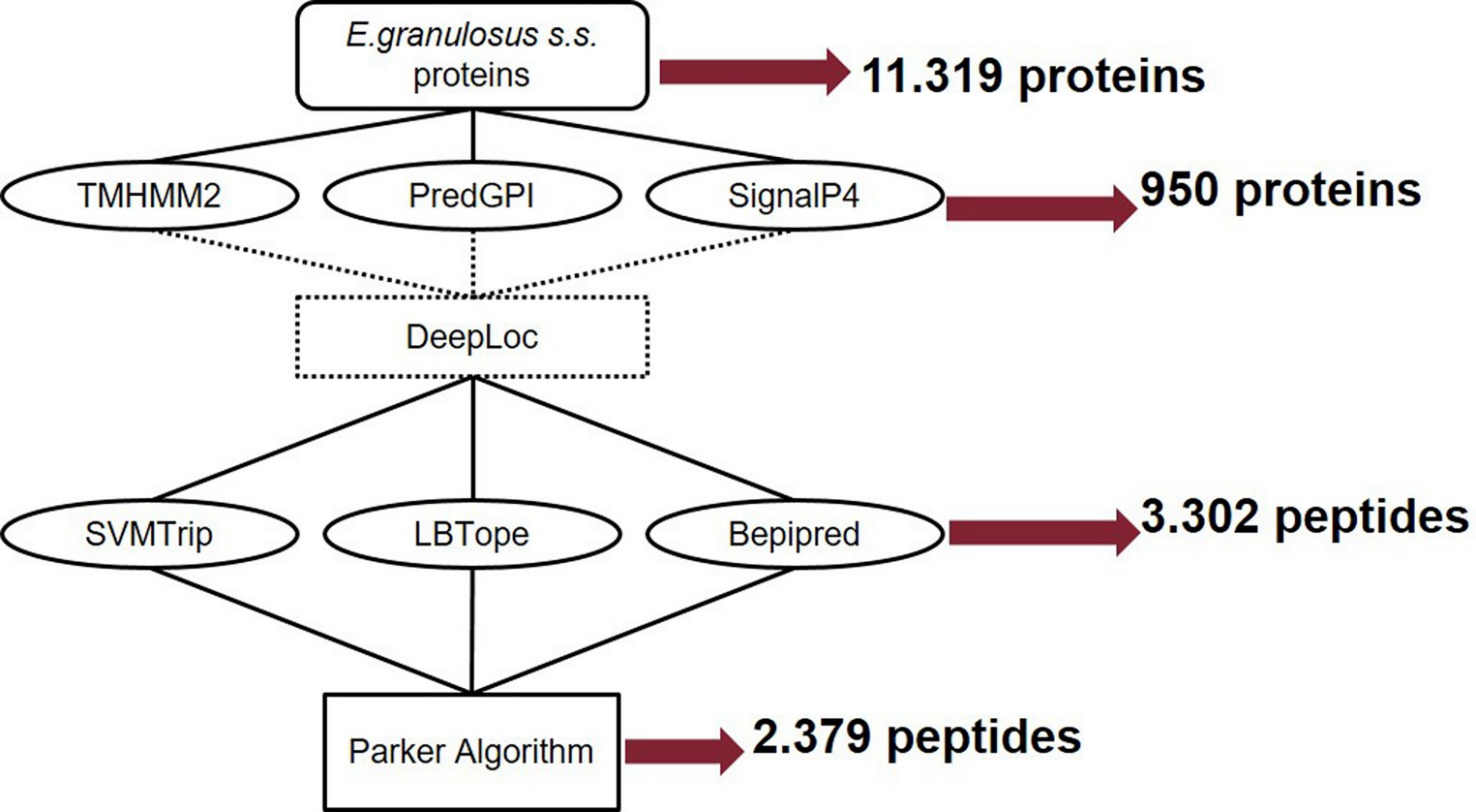
Bioinformatics and experimental workflow to identify most exposed proteins and immunogenic peptides starting from the *E*. *granulosus* s.s. genome. Starting from all proteins predicted from the genome, TMHMM2, SignalP4 and predGPI software identified the most exposed proteins of *E*. *granulosus*. The localization of these proteins was further evaluated using the DeepLoc software and all proteins were retained for the second step of the pipeline to determine specific B-cell antigenic epitopes through Bepipred, SVMTrip e LBTOPE software. All peptides were filtered to avoid redundancy. Finally, peptides were screened according to the hydrophilicity characteristics using the Parker’s algorithm.

We designed and implemented a bioinformatics pipeline to predict, from the genome of *E*. *granulosus* s.s. [12], which proteins could be most likely accessible to the immune system and immunogenic. Starting from all 11,319 proteins predicted from the genome, TMHMM2, SignalP4 [18] and predGPI software identified a total of 950 proteins classified, respectively, as transmembrane proteins, classical secretory proteins, or GPI-anchored proteins (Fig 1) with scores above the set threshold in at least one software.

The localization of these proteins was further evaluated using the DeepLoc software and all proteins were retained for the second step of the pipeline. Peptides resulting as promising based on the statistical analyses were then screened using BLAST v2.2.31 against the non-redundant (nr) database to avoid the inclusion of peptides showing high sequence identity with other parasites (e.g. *Taenia solium*) and thus possibly causing cross-reaction during the immunological tests. The vast majority of these 950 proteins was predicted to be associated to the cell membrane (n = 409), endoplasmic reticulum (n = 215), and extracellular compartment (n = 181). The presence of proteins known for being associated to extracellular vesicles and involved in the host-immune regulation [41–43] such as Calreticulin, Tetraspanin-7, Basement membrane-specific heparan sulfate, Peroxidasin, Antigen B, Neurogenic locus Notch protein, and Basigin was also observed.

The 950 proteins underwent the second screening step of the pipeline to determine, within each protein sequence, specific B-cell antigenic epitopes through Bepipred, SVMTrip e LBTOPE software. These three software identified 225, 1,605 and 1,472 peptide sequences, respectively, for a total of 3,302 peptides predicted as immunogenic from 678 *E*. *granulosus* s.s. proteins. The most redundant peptides were discharged (see methods section). Then, to avoid technical issues during the peptide synthesis due to hydrophobic properties [36], the peptides were ranked according to Parker index selecting the most hydrophilic ones for a total of 2,379 peptides. Finally, we added further six *E*. *granulosus s*.*s*. control peptides by fragmenting them into overlapping 20-mers for a total of 21 peptides (S1 Table).

### 3.3. Peptide microarray

After the bioinformatics selection, a total of 2,400 peptides were synthesized, framed by alternating control spots onto a custom peptide microarray with additional HA (YPYDVPDYAG, 58 spots) and polio (KEVPALTAVETGAT, 58 spots) control peptides as validated reference.

**Fig 2:**
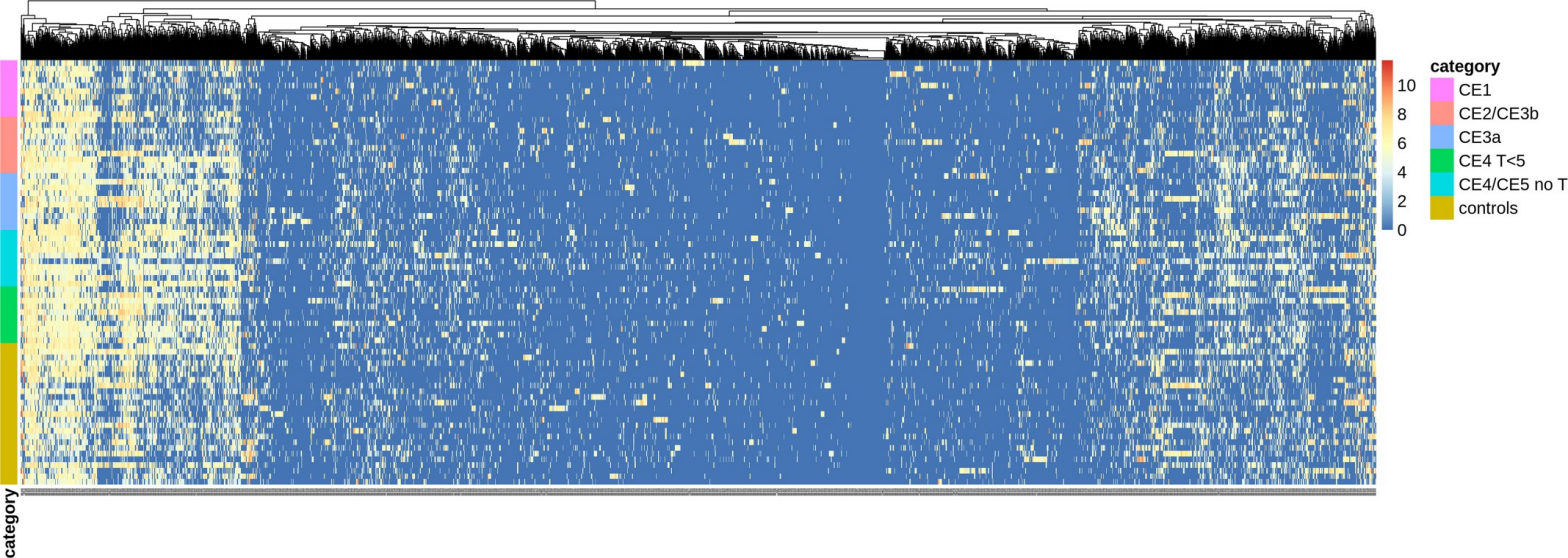
Heatmap summarizing the spot intensities of the peptide microarray for the selected peptides. Microarray reactivities to selected peptides by testing sera from the screening cohort, comprising 50 patients with CE and 25 controls with non-parasitic focal liver lesions. The heatmap depicts the distribution of the spot intensities from the peptide microarray according to the antigenic reaction. The blue and red colors indicate low and high antigenic response respectively. The clustering is generated from the heatmap and it groups the patients (y-axis) and the peptides (x-axis) according to the antigenic response from the microarray. Footnotes: CE, cystic echinococcosis.

Spot intensities from the microarray experiment (Fig 2) were analyzed in two datasets, the first comprising the total 75 serum samples including CE patients and controls, and the second comprising only sera from CE patients comparing active and inactive cysts.

**Fig 3:**
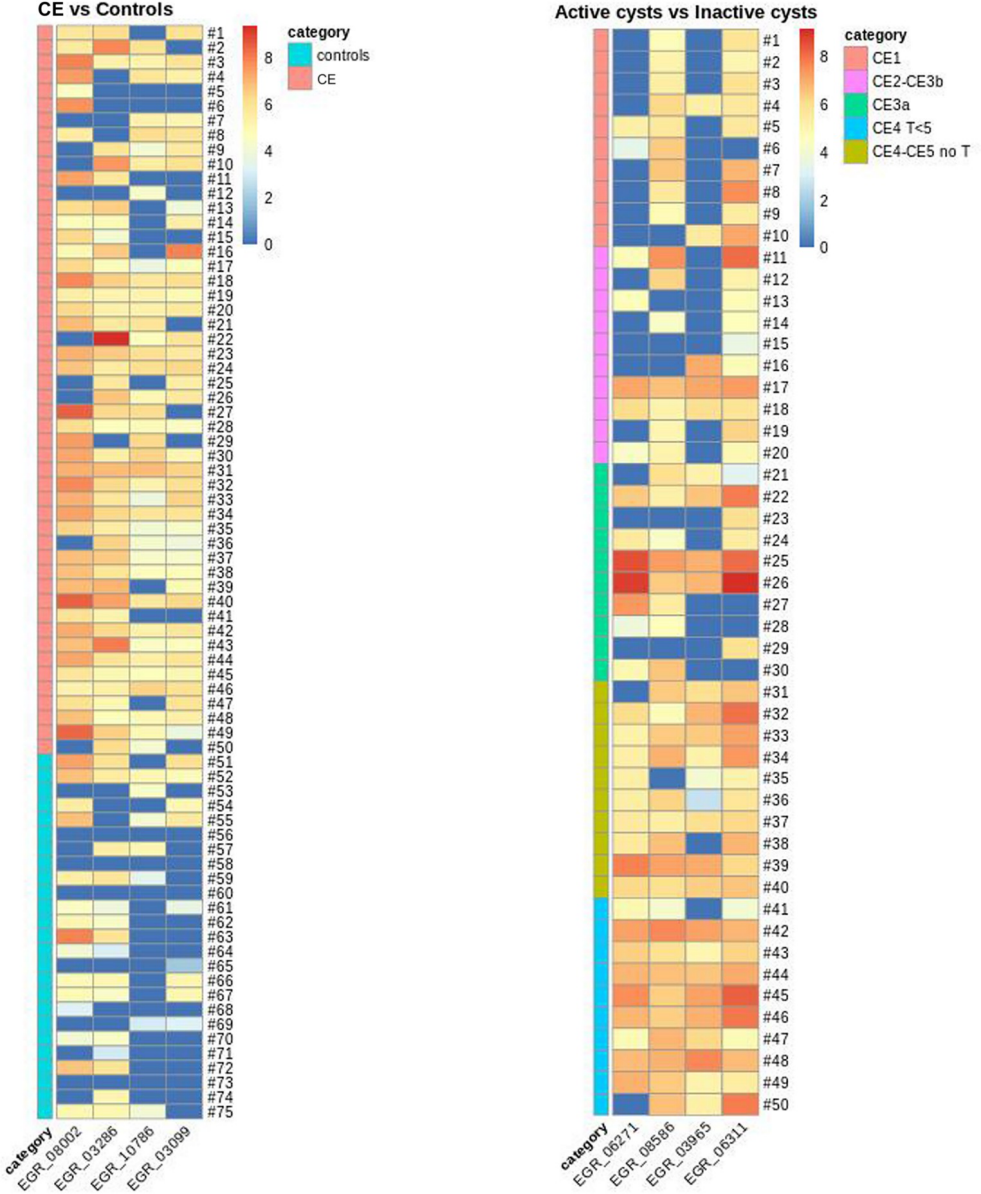
Heatmaps representing the spot intensities of peptide microarray for the selected peptides comparing CE patients and controls or active and inactive CE cysts. **Left panel**. Spot intensities were analyzed comparing CE patients (n = 50) and controls (n = 25) from the screening cohort. **Right panel**. Spot intensities were analyzed within CE patients only comparing patients with active cysts (n = 30) and patients with inactive CE cysts (n = 20) from the screening cohort. Footnotes: CE, cystic echinococcosis.

In order to visualize the microarray spots intensity based on the immunogenic response of each peptide, two heat maps were generated, one for each patient group, using R software with the package “pheatmap” [44] (Fig 3).

The performances of the antigenic peptides as predictors were tested on the dependent variable (presence of CE or presence of active CE cyst) using logistic regression analysis. According to the sensitivity and specificity assessed through the regression model followed by manual curation based on peptide annotations, we selected eight candidates, of which four most promising for the diagnosis of CE infection, and four with potential for discriminating between active and inactive CE cysts. The annotations, as well as the values of sensitivity and specificity predicted for each peptide are summarized in Table 2.

**Table 2:** Sensitivity and specificity of the candidate antigens identified through peptide microarray.

Peptide name	Sequence	Specificity	Sensitivity	AUC	Discrimination test	Annotation
EGR_08002	GEDDEADEDESDEIEDDDGY	72%	76%	0.74	CE vs controls	*Cysticercus cellulosae* specific antigen
EGR_03286	SMTVELEEDFVEDLFDACKD	60%	84%	0.78	CE vs controls	V-type proton ATPase subunit
EGR_03099	RGVLEPIQEELEDAPPLLSQ	72%	78%	0.77	CE vs controls	Potassium voltage-gated channel protein Shal
EGR_10786	LEEHSRLSNLPPNLVIQVDP	80%	76%	0.80	CE vs controls	Nitrogen permease regulator
EGR_08586	CRTHAEYMNYEKRLENVRRS	70%	73%	0.73	Active vs inactive CE cysts	Hypothetical protein
EGR_06311	KCCGMDGWGDFEKLNKSIPA	70%	70%	0.68	Active vs inactive CE cysts	Tetraspanin
EGR_03965	EDPNAALPMMEPLEAMDTEL	90%	70%	0.78	Active vs inactive CE cysts	Hypothetical protein
EGR_06271	PDLEGMLKSMVNKYTATKVV	90%	70%	0.76	Active vs inactive CE cysts	Apoptosis regulator BAX

AUC, Area Under Curve; BAX, *Bcl-2 associated X*.

### 3.4. *E*. *granulosus* EGR_08586 peptide reactivity associates to CE

**Fig 4:**
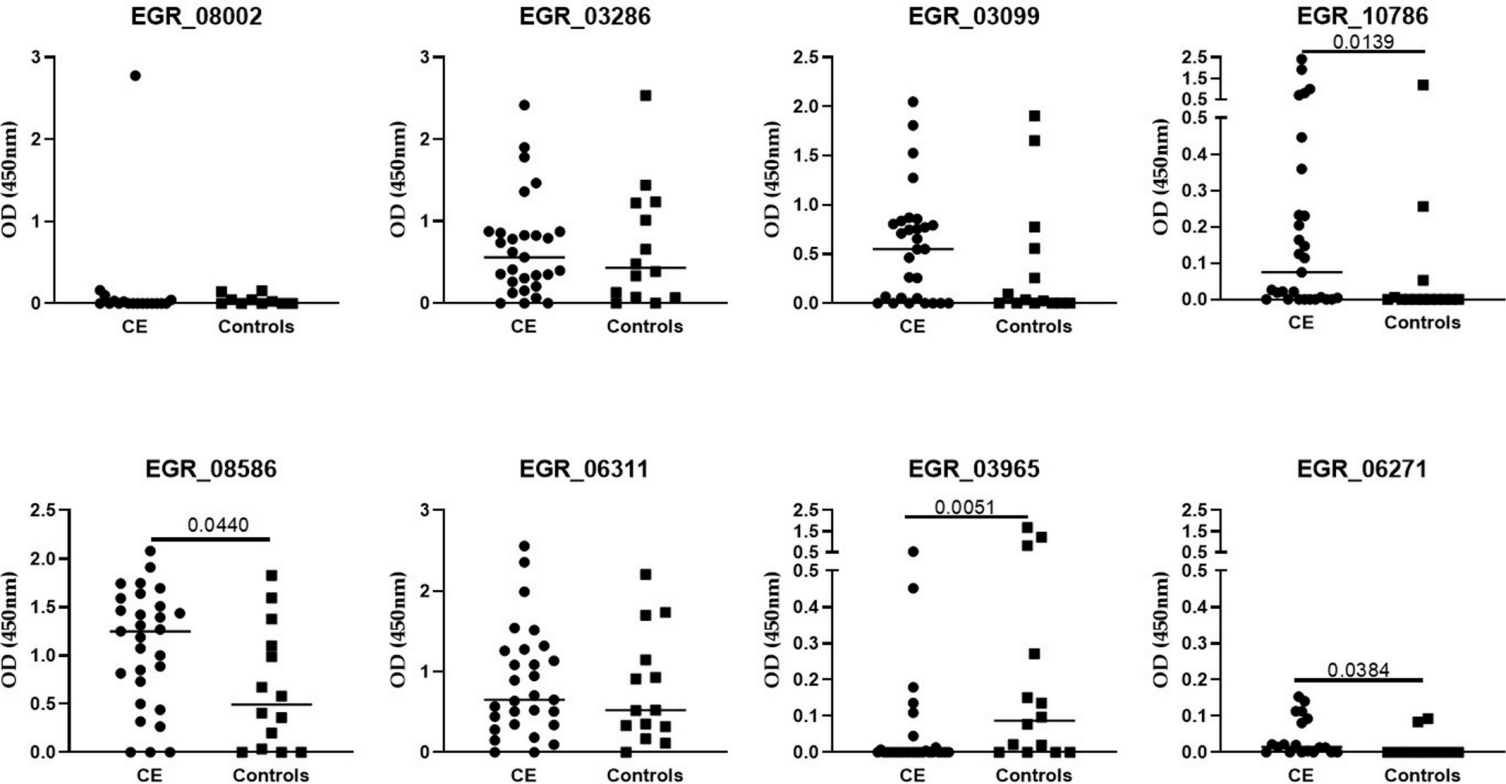
*E*. *granulosus* EGR_08586 peptide reactivity associates to CE. Validation of microarray results using a home-made ELISA by testing sera from the validation cohort comprising 29 patients with CE and 14 controls with non-parasitic focal liver lesions. Peptides obtained from the manual examination of the microarray results and promising for the diagnosis of CE infection (EGR_08002; EGR_03286; EGR_03099; EGR_10786) or for discriminating between active and inactive CE cysts (EGR_08586; EGR_06311; EGR_03965; EGR_06271) were analyzed comparing CE and controls. OD values were subtracted of the background. Horizontal lines represent medians. P value was considered significant if <0.05. Footnotes: CE, cystic echinococcosis; OD, optical density.

A home-made ELISA was set up to validate the microarray results by testing sera from the validation cohort. The analysis of the reactivity to the tested peptides showed that the reactivity to EGR_08586 peptide was significantly higher in the CE group compared to controls (p = 0.044) (Fig 4).

Reactivity to EGR_10786 was also significantly higher in the CE group whereas reactivity to EGR_03965 was higher in controls; however, for both peptides the OD450 values were extremely low and therefore the results difficult to interpret. The reactivity to EGR_03099 was higher in CE patients compared to controls, but not statistically significantly. Finally, the reactivity of the peptides EGR_03286 and EGR_06311 was similar in patients and controls whereas EGR_06271 and EGR_08002 peptides showed no reactivity in both groups (Fig 4). Background analysis of CE and control sera is shown in S1 Fig.

Based on these results we performed a ROC analysis for peptide EGR_08586 only. Significant area under curve (AUC) results were obtained (AUC, 0.69; 95% confidence interval (CI), 0.52–0.86, p = 0.0446). The 0.774 OD450 cut-off maximizing the sensitivity for CE diagnosis, predicted CE with 72.4% sensitivity (95% CI, 54.28%-85.30%) and 64.3% specificity (95% CI, 38.76%-83.66%). The 1.386 OD450 cut-off selected to maximize specificity for CE diagnosis, predicted CE with 41.4% sensitivity (95% CI, 25.51%-59.26%) and 85.7% specificity (95% CI, 60.06%-97.46%). When maximizing both sensitivity and specificity, the 0.99 OD450 cut-off for CE diagnosis showed a 62% sensitivity (95% CI, 44%-77. 31%) and a 71.4 specificity (95% CI, 45.4%-88.3%).

We also evaluated whether the reactivities to the selected peptides could discriminate cysts stages. Due to the small number of samples within each stage-group, we grouped together CE1 and CE2/CE3b (active cysts); therefore, the groups eventually compared were the following: active cysts (CE1 and CE2/3b), transitional cysts (CE3a), inactive CE4/CE5 cysts (CE4/CE5 no therapy), and inactive CE4/CE5 cysts that received therapy in the last 5 years. None of the peptides allows discrimination among the different CE cyst stage/groups (S2 Fig).

## 4. Discussion

Hepatic CE is a neglected and complex disease. Thanks to its unique ability to depict pathognomonic signs of the parasitic cysts in each stage, ultrasonography is the mainstay of the etiological diagnosis of CE cysts, of cyst staging (that guides clinical management), and of follow-up [45]. However, ultrasound machines and expertise in interpreting CE-specific imaging features are not always available. Furthermore, the imaging-based active/inactive aspect of cysts does not perfectly correlate with cyst viability in biological terms. Indeed, CE3a cysts may be biologically viable or not viable [46] and a proportion of inactive CE4/5 cysts may actually be biologically alive as demonstrated by their possible reactivation over time [47–49]. This results in patients requiring years-long follow-up to assess the evolution of infection or the response to treatment over time. Serology has a complementary role since it may allow confirming the diagnosis of CE in case of doubtful imaging [50]. However, the application of serology in the diagnostic workup of CE is not standardized, seroassays are heterogeneous in format and performances, have inadequate diagnostic accuracy especially for CE1 and inactive cysts [6], and are not useful for follow-up since their results do not correlate with cyst’s evolution over time [9]. Therefore, the development of accurate, robust, and easy-to-use, ideally point-of-care, new laboratory tests is urgently needed, to support the etiological diagnosis of the lesions visualized on imaging and the follow-up of patients with CE through identification of the biological viability of the parasite, as well as the development of imaging-independent screening tools.

Currently available seroassays for CE are largely based on crude, variably purified antigens from CE cyst fluid (hydatid fluid) obtained from slaughtered animals, with attending problems in supply and standardization of antigenic composition [8,9]. Several recombinant antigens, which could be produced in large quantity and standardized manner, have been investigated as potential substitutes of the hydatid fluid and its components [9]. However, until now, no recombinant antigen has been able to override current limitations of seroassays for CE, especially concerning sensitivity for early active (CE1) and inactive cysts [6]. Recombinant antigens investigated so far have been largely derived from immunogenic proteins known to be present in high concentration in the hydatid fluid, identified through classic protein-based analysis approaches [10,51]. The identification *of E*. *granulosus* s.l. antigen candidates through peptide microarray starting from the *in-silico* analysis of the parasite genome, the approach carried out in our study, has been implemented only in one previous study [14], which is also the only one so far published on *Echinococcus* spp using this approach [16]. As recently reviewed [16], in the field of human parasitic infections, the microarray technology has been previously explored for the characterization of new diagnostic antigens especially for protozoan infections and, in particular *in silico* prediction of B-cell epitopes, has been mainly applied with positive results in the field of toxoplasmosis [52,53]. Concerning human helminth infections, the peptide microarray approach for antigen discovery with diagnostic scope has been used so far in a very limited number of studies targeting *Schistosoma mansoni* [54], *Onchocerca volvulus* [55], and, as said, *Echinococcus* spp. [14]. In that study, however, List and colleagues [14] did not have access to a fully sequenced genome, thus were only able to start their selection from about 1000 proteins. Additionally, they did not specifically prioritize excreted/secreted proteins, which are known for being potential diagnostic markers thanks to their accessibility to the immune system [56,57]. Finally, only patients with CE cysts in two stages were investigated and healthy blood donors as well as samples from patients with parasitic infections not causing lesions requiring differential diagnosis with CE were used as controls.

In this study, starting from the full *E*. *granulosus* s.s. genome [12], we compiled a database of candidate antigenic peptides having a potential for CE diagnosis based on a multiple-criteria strategy involving the bioinformatics selection of proteins that had the highest probability to be accessible to the immune system, in particular proteins having transmembrane domains and containing a signal peptide [58,59]. Indeed, immunogenic epitopes are present in high density along the transmembrane domains [60]; moreover, the proteins harboring a signal peptide are involved in secretion pathways [61], indicating i) translocation to the cell surface, ii) accessibility by the host immune system [62], and iii) possibility to act as immune-regulators [63]. Considering the antigenic properties of the GPI-anchor and its known role in host-parasite interactions [64,65], GPI-anchor predicted proteins were also added to the analysis. Moreover, the bioinformatics selection was steered to identify epitopes likely recognized by antibodies. The immunogenicity of the selected peptides was tested by microarray using a panel of well-characterized sera obtained from patients with CE cysts in all stages groups, and from clinically meaningful controls, i.e. patients with focal liver lesions potentially requiring differential diagnosis with CE. Our microarray analysis led to the identification of eight antigenic candidates, four of which with an estimated sensitivity of 76%-84% and a specificity of 60%-80% for CE diagnosis vs controls, and four with a sensitivity of 70%-73% and a specificity of 70%-90% for diagnosis of active vs inactive CE cysts. There was a substantial agreement in terms of reactivity of sera to the selected peptides in microarray and in ELISA, as already reported in studies with similar experimental approaches [54]. However, this was overall not reflected in terms of sensitivity and specificity values in the ELISA validation step. Indeed, seven out of eight peptides showed poor accuracy for CE diagnosis or low immunogenicity. Furthermore, none of the candidate peptides was significantly associated to the presence of active or inactive CE cysts. Interestingly, one of these peptides was part of the tetraspanin protein, a membrane protein described as a potential diagnostic candidate for cysticercosis [66] and *Schistosoma japonicum* infection [67]. However, the validation experiment using an independent panel of sera did not further confirm this result for the diagnosis of CE. Only the reactivity to peptide EGR_08586, part of a hypothetical protein, was significantly higher in the CE group compared to controls. Unfortunately, however, the preliminary results of the accuracy of this peptide in ELISA do not favor its potential for clinical use, since (i) its highest possible sensitivity (72%) would be too low to be used for infection screening purposes; (ii) it is not associated specifically to active infection, which would have opened the possibility for its use as an “active CE-only” screening tool; and (iii) its maximum specificity (86%) would also be unsatisfactory and coupled to a too low sensitivity (41%), for its possible use as a confirmatory test.

Altogether, these results show that the identified peptides, used as individual antigens, have at best suboptimal accuracy for the diagnosis of CE and are not useful for cysts stages differentiation. This may be due to a low immunogenicity of these peptides, but technical issues such as a low adsorption onto the ELISA plate due to their intrinsic chemical characteristics may have also occurred. The use of single peptides may also be suboptimal, since it has been suggested that the use a pool of peptides, as opposed to single peptides, might reach higher diagnostic accuracy [68]. Therefore, future studies should focus on the use of combinations of those peptides with predicted good and complementary accuracy for the diagnosis of CE (vs controls) or of active (vs inactive) CE cysts.

This study had several limitations. Firstly, the size of the available samples cohort was relatively low. However, it must be emphasized that many variables are known to influence the results of seroassays [7] and, in the field of echinococcosis, it is exceedingly rare to be able to analyze and obtain results from well-characterized samples from patients with homogeneous clinical characteristics (cyst stage, localization, treatment status), which represents an important value of the study. Secondly, we did not have access to samples from patients with alveolar echinococcosis (AE) caused by *Echinococcus multilocularis*, which can cause liver lesions entering in differential diagnosis with CE, and which shows a very high rate (70–100% [9,69,70] of cross-reactivity with *E*. *granulosus s*.*l*. in seroassays. Three of the eight peptides selected for validation showed *in silico* identity and therefore potential cross-reactivity with *E*. *multilocularis*, including EGR_08586. Therefore, analysis of cross-reactivity and sera from patients with AE will have to be included in any further validation study. Thirdly, a protein, as opposed to a peptide, microarray would have likely allowed identifying different and more performing antigens, since proteins retain their structure allowing complete antibody interactions, including those with conformational epitopes that are recognized by B cells only. However, the use of a protein microarray has been unfortunately not possible in our study, neither was the evaluation of peptide combinations. However, although linear peptides do not provide conformational information, they are recognized by both T and B lymphocytes and can form weak interactions with specific antibodies. Moreover, when the predictions were performed, there were not enough 3D structures of *E*. *granulosus* proteins available in the public databases and *in silico* prediction systems available at the time were still unsatisfactory. Fourthly, here we focused only on three groups of proteins. The choice of these categories was based on their general properties, as mentioned above, which made them theoretically suitable candidates for antigenic properties and secretion in the cyst fluid or in the apical syncytium of the germinal layer, the cellular structure of the parasitic wall. By no means, however, these groups are exhaustive of the protein types with known or potential antigenic properties, neither are surely exposed/released outside the cyst [23,71]. Non-protein molecules were also not included, which could give a more complete picture of the set of antibodies produced against a pathogen [72]. Finally, this is only one of the potential study designs for antigen discovery, which could be applied, while the combination of microarray starting from the pathogen transcriptome or secretome be an alternative approach [16]. Specifically in the field of CE, however, the complex cyst structure, the difficulty in accessing material from cysts in different stages (from either humans, or naturally infected or experimentally infected hosts/animal models), the limitations of *in vitro* CE vesicle culture, and the still limited knowledge of what and how parasite molecules reach the external host environment from the cyst [23,73], make these approaches more difficult. All these aspects surely warrant the attention of further studies, hoping that the attention of funding agency will be driven also on CE, so far excluded by the neglected tropical diseases prioritized for funding.

In conclusion, here we performed bioinformatics analysis and peptide microarray as a discovery approach to identify antigens which could be useful for the diagnosis of CE. Eight candidates were selected and validated. Reactivity to one peptide was significantly higher in CE patients compared to controls but had suboptimal diagnostic accuracy. Nevertheless, our results show that the approach applied in this study is feasible and encourages the scientific community to implement further studies, expanding the scientific methodology and the identification and validation of other antigenic candidates and their combinations, taking advantage also of the *E*. *granulosus* s.s. antigens database compiled in this study.

## Supporting information

S1 Table List of control peptides included in the microarray. (DOCX)

S1 Fig Background analysis of CE and controls sera. Analysis of the background using a home-made ELISA by testing sera from the validation cohort comprising 29 patients with cystic echinococcosis and 14 controls with non-parasitic focal liver lesions. The optical density (OD) read for the wells where no peptides were adsorbed (no Ag) (“peptide-/serum+/secondary antibody+”) was considered as the background and compared with the OD read where peptides were adsorbed (Ag) (“peptide+/serum+/secondary antibody+”). Horizontal lines represent medians. P value was considered significant if <0.05. Footnotes: CE, cystic echinococcosis; OD, optical density; Ag, antigen.
(DOCX)

S2 Fig None of the peptides associate to specific CE cyst stage. Validation of microarray results using a home-made ELISA by testing sera from the validation cohort comprising 29 patients with cystic echinococcosis and 14 controls with non-parasitic focal liver lesions. Peptides resulting from the manual examination of the microarray results promising for the diagnosis of CE infection (EGR_08002; EGR_03286; EGR_03099; EGR_10786) or for discriminating between active and inactive CE cysts are analyzed comparing CE patients with cysts at different stages. The following groups are compared: active cysts (CE1 and CE2/3b), transitional cysts (CE3a), inactive cysts (CE4/CE5 no therapy), and inactive cysts that received therapy in the last 5 years (t<5). OD values were subtracted of the background. Horizontal lines represent medians. P value was considered significant if <0.008.Footnotes: CE, cystic echinococcosis; OD, optical density.
(DOCX)
