# Supplementary material for: Antigen discovery by bioinformatics analysis and peptide microarray for the diagnosis of cystic echinococcosis
Source: PLoS Negl Trop Dis. 2023 Apr 12;17(4):e0011210. doi: 10.1371/journal.pntd.0011210 (PMC10096192; doi:10.1371/journal.pntd.0011210)
Supplement: S1 Table — (DOCX) [file pntd.0011210.s001.docx]

**S1 Table. List of control peptides included in the microarray**

| **Peptide name** | **Sequence** |
| --- | --- |
| EmII/3 | EQKLRELRAQMVEKESDLAD |
| EmII/3 | SDLADMKNKASAYESKIAEL |
| EmII/3 | KASAYESKIAELEMLLQQER |
| EmII/3 | DEVQREVEAQKVAMAKKEAE |
| EmII/3 | KKEAEKAQAEAELRRMREKH |
| EmII/3 | AEKAQAELRRMREKHDAKHK |
| AgB8/1 | KMLGEMKYFFERDPLGQKLV |
| AgB8/1 | GQKLVDLLKELEEVFQMLRK |
| AgB8/1 | LKELEEVFQMLRKKLRTALK |
| AgB8/2 | DPLGQRLVALGNDLTAICQK |
| AgB8/2 | AICQKLQLKIREVLKKYVKN |
| AgB8/2 | REVLKKYVKNLVEEKDDDSK |
| EM13 | QVQNAKNEPFGTPEQLRKIE |
| EM13 | LRKIEDKLRKGIMEEEKTRK |
| EM13 | GIMEEEKTRKAYEEALSSLS |
| PSCCP* | ETIQSLCEHNAALQQKLDEA |
| PSCCP* | KLDEANQSVTEVSVQMKVMQ |
| PSCCP* | SVQMKVMQQHLHTARVAIQS |
| EG19 | EAEAKCLRRPHQRVVKEGEV |
| EG19 | KEGEVSKGDEVDGEDRDDEC |
| EG19 | GDEVDGEDRDDECVGGDEGR |

**Footnote**: Em, *Echinococcus multilocularis*; AgB, Antigen B; PSCCP, protoscolex-specific coiled-coil protein; EG, *Echinococcus granulosus.*
