# Supplementary material for: Antigen discovery by bioinformatics analysis and peptide microarray for the diagnosis of cystic echinococcosis
Source: PLoS Negl Trop Dis. 2023 Apr 12;17(4):e0011210. doi: 10.1371/journal.pntd.0011210 (PMC10096192; doi:10.1371/journal.pntd.0011210)
Supplement: S1 Fig — Analysis of the background using a home-made ELISA by testing sera from the validation cohort comprising 29 patients with cystic echinococcosis and 14 controls with non-parasitic focal liver lesions. The optical density (OD) read for the wells where no peptides were adsorbed (no Ag) (“peptide-/serum+/secondary antibody+”) was considered as the background and compared with the OD read where peptides were adsorbed (Ag) (“peptide+/serum+/secondary antibody+”). Horizontal lines represent medians. P value was considered significant if <0.05. Footnotes: CE, cystic echinococcosis; OD, optical density; Ag, antigen. (DOCX) [file pntd.0011210.s002.docx]

**
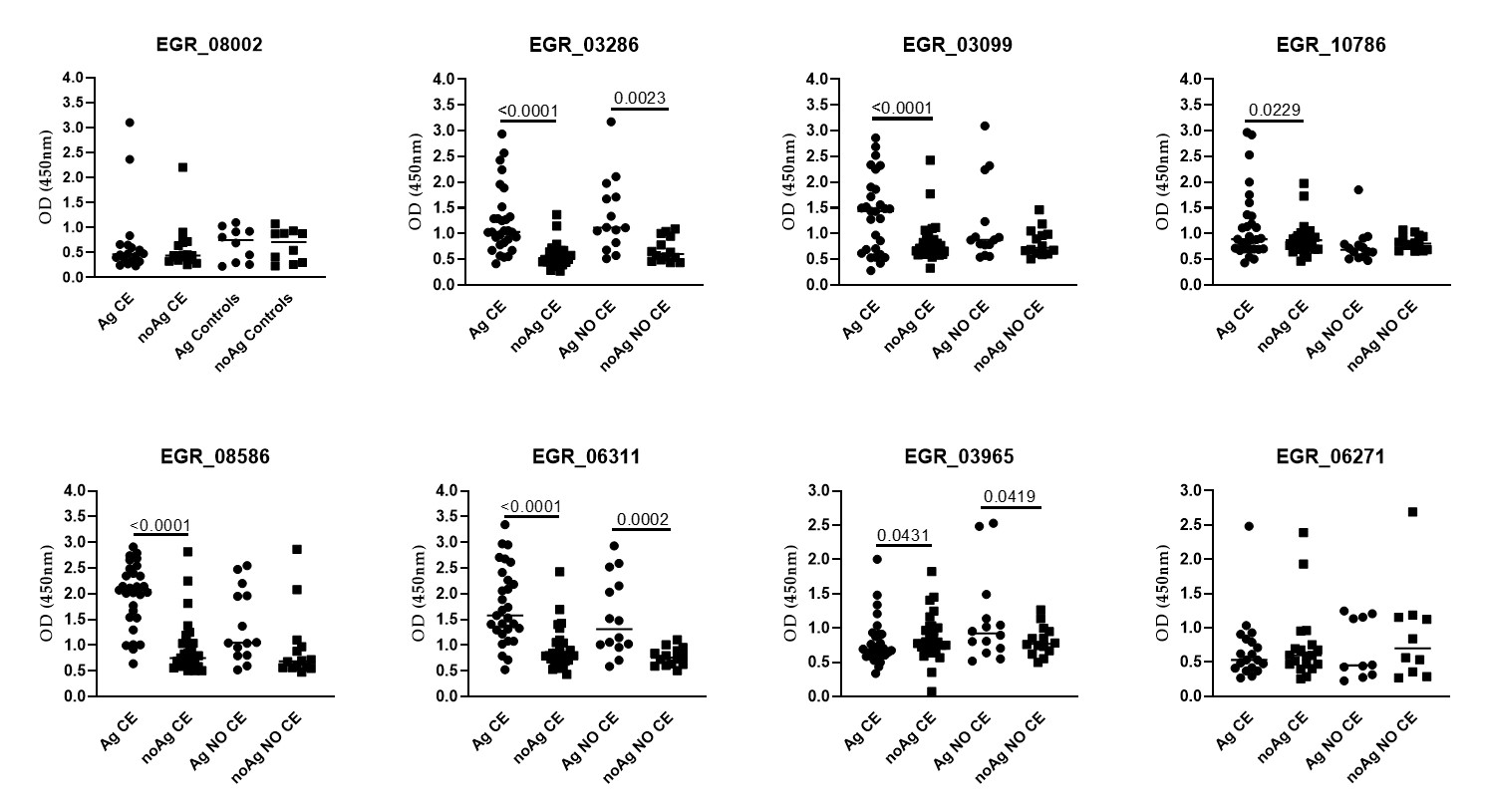
S1 Fig**

**S1 Fig. Background analysis of CE and controls sera.** Analysis of the background using a home-made ELISA by testing sera from the validation cohort comprising 29 patients with cystic echinococcosis and 14 controls with non-parasitic focal liver lesions. The optical density (OD) read for the wells where no peptides were adsorbed (no Ag) (“peptide-/serum+/secondary antibody+”) was considered as the background and compared with the OD read where peptides were adsorbed (Ag) (“peptide+/serum+/secondary antibody+”). Horizontal lines represent medians. P value was considered significant if <0.05. Footnotes: CE, cystic echinococcosis; OD, optical density; Ag, antigen.
