# Supplementary material for: Antigen discovery by bioinformatics analysis and peptide microarray for the diagnosis of cystic echinococcosis
Source: PLoS Negl Trop Dis. 2023 Apr 12;17(4):e0011210. doi: 10.1371/journal.pntd.0011210 (PMC10096192; doi:10.1371/journal.pntd.0011210)
Supplement: S2 Fig — Validation of microarray results using a home-made ELISA by testing sera from the validation cohort comprising 29 patients with cystic echinococcosis and 14 controls with non-parasitic focal liver lesions. Peptides resulting from the manual examination of the microarray results promising for the diagnosis of CE infection (EGR_08002; EGR_03286; EGR_03099; EGR_10786) or for discriminating between active and inactive CE cysts are analyzed comparing CE patients with cysts at different stages. The following groups are compared: active cysts (CE1 and CE2/3b), transitional cysts (CE3a), inactive cysts (CE4/CE5 no therapy), and inactive cysts that received therapy in the last 5 years (t<5). OD values were subtracted of the background. Horizontal lines represent medians. P value was considered significant if <0.008.Footnotes: CE, cystic echinococcosis; OD, optical density. (DOCX) [file pntd.0011210.s003.docx]

**S2 Fig**

**S2 Fig. None of the peptides associate to specific CE cyst stage.** Validation of microarray results using a home-made ELISA by testing sera from the validation cohort comprising 29 patients with cystic echinococcosis and 14 controls with non-parasitic focal liver lesions. Peptides resulting from the manual examination of the microarray results promising for the diagnosis of CE infection (EGR_08002; EGR_03286; EGR_03099; EGR_10786) or for discriminating between active and inactive CE cysts are analyzed comparing CE patients with cysts at different stages. The following groups are compared: active cysts (CE1 and CE2/3b), transitional cysts (CE3a), inactive cysts (CE4/CE5 no therapy), and inactive cysts that received therapy in the last 5 years (t<5). OD values were subtracted of the background. Horizontal lines represent medians. P value was considered significant if <0.008.Footnotes: CE, cystic echinococcosis; OD, optical density.**
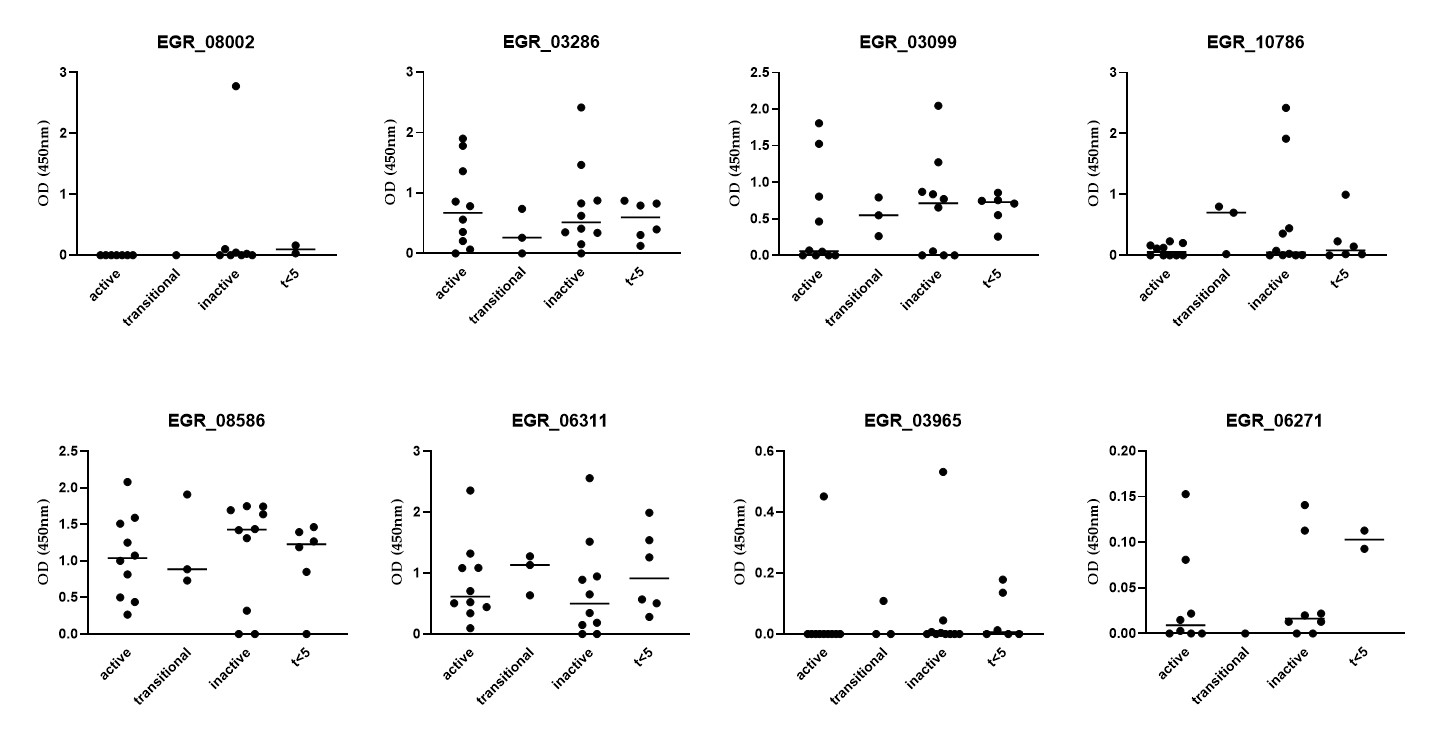
**
